# The Roles of Post-translational Modifications on α-Synuclein in the Pathogenesis of Parkinson’s Diseases

**DOI:** 10.3389/fnins.2019.00381

**Published:** 2019-04-18

**Authors:** Jiaming Zhang, Xiaoping Li, Jia-Da Li

**Affiliations:** ^1^Center for Reproductive Medicine, The First Affiliated Hospital, University of South China, Hengyang, China; ^2^Hunan Key Laboratory of Animal Models for Human Diseases, School of Life Sciences, Central South University, Changsha, China; ^3^Hunan Key Laboratory of Medical Genetics, Center for Medical Genetics, Central South University, Changsha, China

**Keywords:** Parkinson’s disease, α-synuclein, toxicity, post-translational modifications, aggregation

## Abstract

Parkinson’s disease is the second most common neurodegenerative disorder. Although the pathogenesis of Parkinson’s disease is not entirely clear, the aberrant aggregation of α-synuclein has long been considered as an important risk factor. Elucidating the mechanisms that influence the aggregation of α-synuclein is essential for developing an effective diagnostic, preventative and therapeutic strategy to treat this devastating disease. The aggregation of α-synuclein is influenced by several post-translational modifications. Here, we summarized the major post-translational modifications (phosphorylation, ubiquitination, truncation, nitration, *O*-GlcNAcylation) of α-synuclein and the effect of these modifications on α-synuclein aggregation, which may provide potential targets for future therapeutics.

## Introduction

Parkinson’s disease (PD), the second most common neurodegenerative disorder, manifests with resting tremor, bradykinesia, rigidity, postural instability, and gait impairment (Auluck et al., 2010; Tysnes and Storstein, 2017). PD is characterized by loss of dopaminergic neuronal cells in the substantia nigra pars compacta (SNpc) and cytoplasmic deposition of amyloid-like aggregates termed Lewy Bodies (LB) (Forno, 1996; Braak et al., 2003; Shulman et al., 2011).

The major component of LB is α-synuclein aggregates (Spillantini et al., 1997). Furthermore, duplications, triplications, or point mutations in α-synuclein also contribute to some autosomal dominant early-onset PDs and sporadic PDs (Golbe et al., 1990; Polymeropoulos et al., 1997; Kruger et al., 1998; Singleton et al., 2003, 2004; Farrer et al., 2004; Zarranz et al., 2004; Hoffman-Zacharska et al., 2013; Proukakis et al., 2013; Pasanen et al., 2014; Ysselstein et al., 2017). Golbe et al. (1990) identified the α-synuclein A53T mutation in a PD patient. Several other mutations have been identified since then, such as A30P, A18T, A29S, E46K, H50Q, G51D, and A53E.

The contribution of α-synuclein in the pathogenesis of PD has been extensively studied in a variety of animal models, including mice, Drosophila, and *Caenorhabditis elegans*. Transgenic mice or flies overexpressing WT, A30P or A53T α-synuclein show motor deficits and neuronal inclusions (Feany and Bender, 2000; Kahle et al., 2000; Masliah et al., 2000; van der Putten et al., 2000; Maguire-Zeiss et al., 2005; Lelan et al., 2011; Lin et al., 2012). The α-synuclein aggregates in dopaminergic neurons are found in WT, A30P, or A53T human α-synuclein transgenic nematodes *C. elegans* (Kuwahara et al., 2006). Overexpression of human α-synuclein in *C. elegans* causes age- and dose-dependent dopaminergic neurodegeneration (Cao et al., 2005; Hamamichi et al., 2008).

α-Synuclein also undergoes extensive post-translational modification (PTM), which influence the aggregation and/or cytotoxicity. PTMs may mediate the environmental factors on the pathogenesis. In this review, we will summarize physiological and pathological roles of α-synuclein, emphasizing the involvement of PTMs.

## Structure of α-Synuclein

In humans, α-synuclein is a member of synuclein family, which includes α-synuclein, β-synuclein, and γ-synuclein (Lashuel et al., 2013). α-Synuclein, a 140-amino acid protein, is composed of three distinct domains. The N-terminus (1–60 residues) contains four imperfect KTKEGV motif repeats. The central hydrophobic domain of α-synuclein (61–95 residues), also known as the non-amyloid component (NAC), is crucial for its aggregation (Giasson et al., 2001). The C-terminus (96–140 residues) is enriched in acidic residues and is the major phosphorylation site (Uversky and Eliezer, 2009).

α-Synucleins purified from bacterial or mouse tissues under denaturing conditions are ‘natively unfolded’ monomers of about 14 kDa (Weinreb et al., 1996). It may acquire α-helical secondary structure upon binding to lipid vesicles (Davidson et al., 1998; Eliezer et al., 2001). Bartels et al. (2011) found that endogenous α-synuclein under non-denaturing conditions form a folded tetramer and non-crosslinked monomer in all cells, plus some putative dimers in the HeLa, HEK, and red blood cells. They further showed that very few native human α-synuclein tetramers form aggregation, whereas recombinantly expressed monomers readily aggregated into amyloid-like fibrils *in vitro* (Bartels et al., 2011).

## Function of α-Synuclein

α-Synuclein is mainly expressed at presynaptic terminals and has been implicated in numerous cellular processes (Adamczyk et al., 2005). However, the exact physiological function of α-synuclein is still unclear. Under physiological conditions, α-synuclein may be involved in the compartmentalization, storage, and recycling of neurotransmitters (Allen Reish and Standaert, 2015).

Soluble *N*-ethylmaleimide-sensitive factor attachment protein receptor (SNARE) proteins are crucial for release of neurotransmitters at the neuronal synapse, vesicle recycling, and synaptic integrity (Goda, 1997; Gerst, 1999). Burre et al. (2010) demonstrated that α-synuclein acts as a molecular chaperone to assist the folding and refolding of SNARE proteins. α-Synuclein directly binds to the SNARE protein synaptobrevin-2 and promote the formation of SNARE-complex (Burre et al., 2010). Moreover, α-synuclein is also involved in the dynamics of synaptic vesicles (SVs) trafficking to control the amount of vesicles docked at the synapses during neurotransmitter release (Burre, 2015). As a result, α-synuclein null mice exhibit accelerated recovery of neurotransmitter release when presented with multiple stimuli. Depletion of α-synuclein from rodent hippocampal neurons also induces a significant loss of undocked SVs (Cabin et al., 2002).

## Aggregation of α-Synuclein

Aggregates of α-synuclein are the major component of Lewy body, the pathological marker of PD, dementia with Lewy bodies and Lewy body variant of Alzheimer’s disease (Spillantini et al., 1997, 1998). The aggregation of α-synuclein is formed in three steps. The first step is the rate-limiting step, in which the soluble unstructured monomeric species were converted into partially soluble oligomers when nucleation-dependent chain polymerization occurs. Then, the oligomers aggregate into insoluble mature fibrils. At last, the amyloid fibrillar aggregates are formed (Harper et al., 1997; Walsh et al., 1997; Lambert et al., 1998). Miake et al. (2002) have shown that α-synuclein filaments assembled *in vitro* or extracted from multiple system atrophy (MSA) brains are insoluble to detergents and partially resistant to proteinase K (PK) digestion. Variable amounts of neuritic PK-resistant α-synuclein have been detected in the striatum of all the LB disease cases. PK resistance of α-synuclein may be useful for the development of biomarkers of LB diseases (Neumann et al., 2004).

Both fibrils and oligomers have been shown to display toxicity. Peelaerts et al. (2015) showed that α-synuclein fibrils can lead to progressive motor impairment and cell death. Lots of studies have suggested that amyloids associated with neurodegenerative diseases spread in a prion-like fashion. Fibrillar α-synuclein assemblies seed the aggregation of monomeric α-synuclein *in vitro* and spread from one cell to another in cell cultures and animal models (Wood et al., 1999; Desplats et al., 2009; Hansen et al., 2011). Multiple lines of evidence have also suggested that oligomeric species of α-synuclein are toxic. In this review, we mainly summarized the evidence supporting the toxicity of α-synuclein oligomers in PD and possible mechanisms for this toxicity.

## Toxicity of α-Synuclein

α-Synuclein aggregates may cause cytotoxicity through several pathways, such as mitochondrial dysfunction, endoplasmic reticulum (ER) stress, proteasome system dysfunction, phagocytosis and inflammatory response in microglia, membrane damage, and synaptic dysfunction.

### Mitochondrial Dysfunction

The loss of dopaminergic neurons is a major pathological feature of PD patient. Dopaminergic neurons are particularly sensitive to mitochondrial dysfunction due to their high energy demands and increased oxidative stress (Ryan et al., 2015). Both the monomer and oligomer of α-synuclein show toxicity to mitochondria. The translocase of the outer membrane (TOM) 20 receptors are important for the mitochondrial protein import. α-Synuclein can inhibit the protein import of mitochondria by binding to TOM20 (Di Maio et al., 2016). The voltage-dependent anion channel (VDAC) is the major channel of the mitochondrial outer membrane, which controls most of the metabolite fluxes in and out of the mitochondria. Rostovtseva et al. (2015) showed that monomeric α-synuclein reversibly block VDAC in a highly voltage-dependent manner.

α-Synuclein oligomers cause mitochondria fragmentation in a dopaminergic cell line SH-SY5Y (Plotegher et al., 2014). α-Synuclein oligomers decreased the retention time of exogenously added calcium, promoted calcium-induced mitochondrial swelling and depolarization. α-Synuclein oligomers also accelerated cytochrome C release, which cause the apoptosis of dopaminergic neurons (Luth et al., 2014).

### Endoplasmic Reticulum Stress

Endoplasmic reticulum is responsible for the synthesis, modification, and delivery of proteins to their target sites within the secretory pathway and the extracellular space. Disruption of any of these processes may cause ER stress (Hampton, 2000). The folding-incompetent proteins can cause ER stress and an ER stress response, called unfolded protein response (UPR). UPR is the biochemical basis for many ER storage diseases (Schroder and Kaufman, 2005). Castillo-Carranza et al. (2012) showed that α-synuclein oligomers induced ER stress in SH-SY5Y cells. Colla et al. (2012) found that accumulation of the toxic α-synuclein oligomers are temporally and spatially linked to the induction of chronic ER stress in the α-synuclein transgenic mice.

### Proteasome System Dysfunction

Ubiquitin proteasome system is a highly regulated mechanism of intracellular protein degradation and turnover (Tanaka and Chiba, 1998). PD patients have a vulnerable proteasomal function in the substantia nigra, which may be due to the inhibition of α-synuclein oligomers on the proteasomal system (McNaught and Jenner, 2001; McNaught et al., 2001, 2002, 2003). Indeed, α-synuclein is co-localized with ubiquitin and 20S proteasomal components in Lewy bodies. The α-synuclein oligomers may directly bind to the 20S proteasome. Binding of α-synuclein oligomers to the proteasome inhibits the chymotrypsin-like proteasomal activity of the 20S proteolytic particle (Lindersson et al., 2004). Interestingly, A53T α-synuclein oligomers impaired the proteasomal activity in PC12 cells, which can be reversed by Congo Red, an inhibitor of α-synuclein oligomerization (Emmanouilidou et al., 2010).

### Phagocytosis and Inflammatory Response in Microglia

Microglia are the resident macrophage cells in the central nervous system (CNS), involved in chemotaxis, phagocytosis, and secretion of a variety of cytokines and proteases. Microglia have a close relationship with the pathogenesis of PD (Sanchez-Guajardo et al., 2015; Ferreira and Romero-Ramos, 2018). Park et al. (2008) found that microglial phagocytosis is enhanced by extracellular monomeric α-synuclein but inhibited by the aggregated α-synuclein. The inflammatory response in microglia is activated by Toll-like receptor 2 (TLR2) (Stirling et al., 2014). Kim et al. (2013) showed that extracellular α-synuclein released from neuronal cells is an endogenous agonist for TLR2.

### Membrane Damage

The cell membrane is an important barrier to prevent extracellular substances from entering the cell, which ensures the relative stability of the intracellular environment and enables various biochemical reactions to run in an orderly manner. Membrane integrity is essential for the basic function of all cell types. Dysfunctional membranes can also lead to abnormal calcium homeostasis. α-Synuclein has been shown to undergo accelerated aggregation at membrane surfaces when incubated with synthetic or natural phospholipid vesicles or supported lipid bilayers, presumably because the two dimensional surface of the membrane increases the probability of molecular interactions needed for oligomerization (Haque et al., 2010). Danzer et al. (2007) showed some types of α-synuclein oligomers induced cell death via disruption of cellular calcium influx by a presumably pore-forming mechanism. Angelova et al. (2016) further confirmed that α-synuclein interacts with membranes to affect Ca2+ signaling in a structure-specific manner and the oligomeric β-sheet-rich α-synuclein species ultimately leads to Ca2+ dysregulation.

Several approaches have been developed to alleviate the α-synuclein-induced membrane damage. Endosulfine-α, which can bind specifically to membrane-associated α-synuclein, alleviates dopaminergic cell death by interfering with the formation of neurotoxic α-synuclein oligomers at the membrane surface (Ysselstein et al., 2017). A novel compound NPT100-18A, which can displace α-synuclein from the membrane, can also reduce a-synuclein toxicity (Wrasidlo et al., 2016).

### Synaptic Dysfunction

Synaptic dysfunction is an early pathological feature of PD (Schulz-Schaeffer, 2010). SNARE complex is required for SV fusion. α-Synuclein oligomers prevent the formation of the SNARE complex by binding to synaptobrevin (Choi et al., 2013).

Axonal transport, which relies on the microtubule (MT) network, is fundamental for the maintenance of neuronal homeostasis (Goldstein et al., 2008). Prots et al. (2013) showed that α-synuclein oligomers significantly inhibited MT assembly. 3,4-Dihydroxyphenylacetaldehyde (DOPAL) is a catabolite generated from dopamine by monoamine oxidase (Burke et al., 2003; Goldstein et al., 2011). It has been shown that DOPAL can cause α-synuclein oligomerization *in vitro* and in cell models (Burke et al., 2008; Lima et al., 2018). Plotegher et al. (2017) showed that this kind of α-synuclein-DOPAL oligomers can permeabilize cholesterol-containing lipid membranes mimicking SVs *in vitro*, which suggests that the synergistic effect of α-synuclein and DOPAL accumulation in DA neurons may lead to the formation of oligomers, negatively impacting the structure and function of SVs.

Vesicles for synaptic release are produced by the Golgi apparatus. The dysfunction of Golgi can lead to abnormity of synaptic function. Gosavi et al. (2002) showed that over-expression of α-synuclein in COS-7 cells caused Golgi fragmentation. As mentioned previously, pore-like oligomers of α-synuclein could also rupture SVs, leading to decreased neurotransmitter release, as well as permeabilization of cell membranes, which could result in Ca2+ influx and excitotoxicity (Danzer et al., 2007).

## Post-Translational Modifications of α-Synuclein

α-Synuclein is subjected to extensive post-transcriptional modifications (PTMs), including phosphorylation, ubiquitination, nitration, truncation, and *O*-GlcNAcylation. PTMs of α-synuclein may influence its toxicity and aggregation.

### Phosphorylation

α-Synuclein within LB can be phosphorylated at serine 129 and 87 (S129-P, S87-P) (Hasegawa et al., 2002; Anderson et al., 2006; Paleologou et al., 2010). S129-P has emerged as a defining hallmark of PD and related synucleinopathies. Feany and Bender (2000) showed that α-synuclein is also phosphorylated at tyrosine 125, 133, and 136 (Y125-P, Y133-P, and Y136-P) (Ellis et al., 2001; Nakamura et al., 2001; Ahn et al., 2002; Negro et al., 2002; Takahashi et al., 2003). The kinases that mediate phosphorylation at Y125 of α-synuclein are still unknown. Hejjaoui et al. (2011) have developed a semi-synthetic strategy that enables the site-specific introduction of single phosphorylation at Y125. They showed that phosphorylation at Y125 does not affect the fibrillization of α-synuclein (Burai et al., 2015). The impact of the phosphorylation at tyrosine 133 and 135 on α-synuclein aggregation is still unknown.

A number of kinases have been shown to phosphorylate α-synuclein at S129 *in vitro*, including casein kinase I (CKI), casein kinase II (CKII), the G protein-coupled receptor kinases (GRK), LRRK2, and polo-like kinases (PLK) (Okochi et al., 2000; Pronin et al., 2000; Inglis et al., 2009).

Fujiwara et al. (2002) showed that phosphorylation of S129 in α-synuclein by CKII promotes *in vitro* fibrillation. Smith et al. (2005) indicated that phosphorylation at S-129 by CKII promotes the formation of cytoplasmic inclusions in some cell culture models.

Phosphorylation of α-synuclein at S-129 by GRK2 was reported to be toxic. Feany and Bender (2000) have studied the phosphorylation of α-synuclein in Drosophila. They showed that co-expression of Drosophila GRK2 with α-synuclein enhances the formation of α-synuclein oligomers and accelerates neuronal loss, as compared to the Drosophila expressing α-synuclein alone (Feany and Bender, 2000).

α-Synuclein phosphorylation at S129 is largely reduced in PLK2-/- transgenic mice, supporting the involvement of PLK in α-synuclein phosphorylation *in vivo* (Inglis et al., 2009). PLK2-induced phosphorylation has no effect on the aggregation of α-synuclein. Nevertheless, Oueslati et al. (2013) showed that PLK2 binds directly to α-synuclein in an ATP-dependent manner and regulates α-synuclein selective clearance via the lysosome–autophagic degradation pathway, which suggests a neuroprotective role of PLK2 against PD pathology.

### Ubiquitination and Sumoylation

The core of LBs is immunoreactive for both α-synuclein and ubiquitin proteins and is surrounded by a rim of α-synuclein (Gomez-Tortosa et al., 2000). However, the major α-synuclein species in LBs is mono-, di-, and tri-ubiquitinated, suggesting the involvement of ubiquitination in the pathophysiologic properties of α-synuclein (Hasegawa et al., 2002; Sampathu et al., 2003; Tofaris et al., 2003; Nonaka et al., 2005). The ubiquitination of α-synuclein is correlated with three E3 ubiquitin-protein ligases: C-terminal U-box domain of co-chaperone Hsp70-interacting protein (CHIP), seven in absentia homolog (SIAH) and neuronal precursor cell-expressed, developmentally down-regulated gene 4 (Nedd4) (Liani et al., 2004; Shin et al., 2005; Tofaris et al., 2011).

The mammalian homologs of Drosophila seven in absentia (SIAH-1 and SIAH-2) gene have been characterized as a family of RING-type E3 ligases (Wheeler et al., 2002). Both *in vivo* and *in vitro* data showed that ubiquitination of α-synuclein by SIAH promotes the formation of inclusions. Rott et al. (2008) showed that ubiquitination of α-synuclein *in vitro* by SIAH promotes the formation of higher molecular weight α-synuclein. They then used electron microscopy to show that α-synuclein ubiquitinated by SIAH formed more aggregates (Rott et al., 2008). Lee et al. (2008) also showed that SIAH-1 or SIAH-2-mediated ubiquitination enhances the aggregation of α-synuclein and formation of α-synuclein-positive inclusion in PC12 cells and SH-SY5Y human neuroblastoma (Lee et al., 2008).

CHIP is a multidomain chaperone, utilizing both a tetratricopeptide/Hsp70 binding domain and a U-box/ubiquitin ligase domain to recognize misfolded proteins (Demand et al., 2001; Murata et al., 2001). CHIP is able to mono- and poly-ubiquitinate α-synuclein (Kalia et al., 2011). Shin et al. (2005) showed that CHIP colocalizes with α-synuclein in Lewy bodies and also in a cell culture model of α-synuclein inclusions. Overexpression of CHIP inhibits α-synuclein aggregation and increases α-synuclein degradation in cell culture. Interestingly, they also indicated that CHIP can regulate α-synuclein degradation both via the proteasomal degradation pathway and the lysosomal degradation pathway (Shin et al., 2005). A study from Tetzlaff et al. (2008) showed that CHIP selectively reduced α-synuclein oligomerization and toxicity in a tetratricopeptide domain-dependent, U-box independent manner by specifically degrading toxic α-synuclein oligomers.

Nedd4 is a HECT-domain E3 that functions at the plasma membrane in the turnover of a number of membrane-associated proteins. Tofaris et al. (2011) showed that Nedd4 can act as an E3 for α-synuclein. They demonstrated that Nedd4 directly binds to α-synuclein in brain and cell extracts and promotes the degradation of endogenous α-synuclein by lysosomes (Tofaris et al., 2011). They further found that Nedd4-mediated degradation protects against α-synuclein-induced toxicity in the Drosophila and rodent models of Parkinson’s disease (Davies et al., 2014). Nedd4-1-linked Lys-63 ubiquitination was demonstrated to specify the fate of extrinsic and *de novo* synthesized α-synuclein by facilitating their targeting to endosomes (Sugeno et al., 2014). In yeast ubiquitin ligase, the Nedd4 ortholog Rsp5 is a key enzyme involved in the degradation of abnormal or unfavorable proteins. Wijayanti et al. (2015) have isolated novel hyperactive forms of Rsp5 that alleviate α-synuclein toxicity, by enhancing the clearance of α-synuclein, including the processes of interaction, ubiquitination, and degradation.

The site-specific effects of ubiquitination on aggregation and clearance have been studied using a semi-synthetic strategy (Hejjaoui et al., 2011; Abeywardana et al., 2013). Monomeric ubiquitination of α-synuclein at K6 was shown to resist fibril formation when compared to unmodified protein (Hejjaoui et al., 2011); Ubiquitination of α-synuclein at K10 and K23 readily form fibrils; Ubiquitination of α-synuclein at K6, K12, and K21 moderately inhibit the formation of fibrils; Ubiquitination of α-synuclein at K32, K34, K43, and K96 displayed no fibril formation, suggesting a strong inhibitory effect (Meier et al., 2012). Haj-Yahya et al. (2013) have incorporated K48-linked di- or tetra-Ub chains onto the side chain of Lys12 of α-synuclein and demonstrated that the length of the Ub chain plays an important role in regulating α-synuclein fibril formation and clearance.

α-Synuclein is also conjugated to small ubiquitin-like modifier (SUMO) at lysines. Rott et al. (2017) demonstrated that α-synuclein is SUMOylated by PIAS2, and SUMOylated α-synuclein and PIAS2 are markedly elevated in the substantia nigra of PD brains. Further, Lewy bodies are positive for both SUMO1 and PIAS2. They found that SUMOylation increases α-synuclein aggregation by two self-reinforcing mechanisms. First, SUMOylation by PIAS2 directly promotes the aggregation of α-synuclein. Second, SUMOylation impairs α-synuclein ubiquitination and prevents α-synuclein degradation. Therefore, SUMOylation blockers may provide a strategy to prevent intracellular α-synuclein aggregation (Rott et al., 2017). However, Krumova et al. (2011) showed that sumoylation inhibits α-synuclein aggregation and toxicity. *In vitro* study demonstrated that SUMOylation at K102 of α-synuclein results in more pronounced inhibition of aggregation than the corresponding modification at K96 (Abeywardana and Pratt, 2015).

### Truncation

Besides full-length α-synuclein, there exist small amounts of various truncated species with apparent molecular masses of 10–15 kDa in the LBs (Baba et al., 1998; Crowther et al., 1998; Campbell et al., 2001). It is estimated that about 15% α-synuclein in LBs is truncated. And the C-terminally truncated α-synuclein may act as effective seeds to accelerate the aggregation of the full-length protein.

The carboxyl-terminal-truncated α-synuclein produced by aberrant proteolysis, is found in association with α-synuclein aggregates (Tofaris et al., 2003). Tofaris et al. (2003) investigated the effects of truncation by generating both transgenic Drosophila and transgenic mice expressing human α-synuclein. They found that the truncated form of α-synuclein (1–120) increased accumulation of high molecular weight α-synuclein species, and enhanced neurotoxicity *in vivo* (Periquet et al., 2007). They showed that the striatal dopamine levels are reduced and the transgenic mice showed a progressive reduction in spontaneous locomotion and an increased response to amphetamine (Tofaris et al., 2006). Hoyer et al. (2004) used recombinant proteins and showed that the fragments (1–110; 1–119; 110–140) promoting nucleation seed the aggregation of full-length α-synuclein. Murray et al. (2003) also showed that the truncated α-synuclein variants, 1–89, 1–102, 1–110, 1–120, and 1–130 aggregated more rapidly than the full-length protein.

Several enzymes have been implicated in the truncation of α-synuclein, including calpain I, Neurosin, Cathepsin D, and Matrix metalloproteinase 3 (Iwata et al., 2003; Mishizen-Eberz et al., 2005; Sevlever et al., 2008; Choi et al., 2011).

Since α-synuclein is predominantly localized to the pre-synaptic terminal, it may be a substrate for soluble or membrane-associated proteases such as the calcium-activated neutral protease calpain I. Mishizen-Eberz et al. (2003) demonstrated that Calpain I cleaves wild-type α-synuclein predominantly after amino acid 57 and within the NAC region (73, 74, and 83). Calpain-mediated processing of soluble α-synuclein inhibits fibrillization, while processing of fibrillar α-synuclein promotes further aggregation (Mishizen-Eberz et al., 2005).

Neurosin, a serine protease predominantly expressed in the CNS, is presumed to play an important role in the degradation of α-synuclein (Iwata et al., 2003). Neurosin cleaves α-synuclein after amino acid 80 and 97. Cleavage of α-synuclein after 80 by neurosin may inhibit the polymerization; however, the fragment cleaved after 97 has a stronger propensity to polymerize than non-processed α-synuclein (Kasai et al., 2008).

### Nitration

Oxidative injury has been implicated in the pathogenesis of PD (Schapira and Jenner, 2011; Bose and Beal, 2016). The action of oxygen and nitric oxide and their products, especially peroxynitrite, leads to the nitration of tyrosine residues in proteins. Giasson et al. (2000) first showed that a-synuclein is nitrated when present in the major filamentous and in the insoluble fractions of affected brain regions of synucleinopathies. All four tyrosine residues in α-synuclein (Y39, Y125, Y133, and Y136) are susceptible to nitration (Sevcsik et al., 2011; Burai et al., 2015).

Danielson et al. (2009) showed that nitration of Y-39 accelerates the oligomerization of α-synuclein, and a mutation in this residue leads to high levels of fibrilization.

Hodara et al. (2004) showed that monomeric or dimeric forms of nitrated α-synuclein accelerate the fibril formation and seed the fibrillation of non-modified α-synuclein. On the other hand, nitrated α-synuclein oligomers inhibit the fibril formation (Hodara et al., 2004).

Through site-specific incorporation of 3-nitrotyrosine at different regions of α-synuclein, Burai et al. (2015) indicated that different site-specifically nitrated α-synuclein species exhibit distinct aggregation properties. They further showed that intermolecular interactions between the N- and C-terminal regions of α-synuclein play critical roles in mediating nitration-induced α-synuclein oligomerization (Burai et al., 2015).

### *O*-GlcNAcylation

*O*-GlcNAcylation is a dynamic biochemical process, in which *N*-acetylglucosamine (GlcNAc) from uridine 5′-diphospho-*N*-acetylglucosamine (UDP-GlcNAc) is transferred to the serine and threonine residues of proteins by *O*-GlcNAc transferase (OGT) and removed by *O*-GlcNAcase (OGA) (Hart et al., 2007). More than 1,000 proteins can be modified by *O*-GlcNAc, including molecular chaperones, transcription factors, RNA polymerase II, nucleoporin, RNA binding proteins, kinases, and cytoskeletal proteins (Hardiville and Hart, 2014). *O*-GlcNAcylation has identified threonine (T) residue 33, 34, 54, 59, 64, 72, 75, 81, and 87 of α-synuclein isolated from mouse and human samples (Wang et al., 2009, 2010, 2017; Alfaro et al., 2012; Morris et al., 2015).

To understand the effect of *O*-GlcNAcylation on the aggregation of α-synuclein, Marotta et al. (2015) synthesized a peptide of α-synuclein comprising residues 68–77, in which the T72 is *O*-GlcNAcylated. As compared with the unmodified peptide, the *O*-GlcNAcylated peptide inhibits full-length α-synuclein fibrillization. They further synthesized a full-length α-synuclein, with *O*-GlcNAcylation at T72. *O*-GlcNAcylation at T72 completely blocks the formation of both fiber and oligomer aggregates *in vitro*. They synthesized a full-length α-synuclein with *O*-GlcNAcylation at S87, which still aggregates but with slower kinetics than the unmodified protein (Lewis et al., 2017). Recently, Levine et al. (2019) showed that several of the *O*-GlcNAc sites inhibit the toxicity of extracellular α-synuclein fibers that are likely culprits in the spread of PD. They also demonstrated that *O*-GlcNAcylation can inhibit the aggregation of an aggressive mutant of α-synuclein.

To study the functional consequences of enzymatic *O*-GlcNAcylation of α-synuclein, we co-expressed a shorter form of OGT (sOGT) and α-synuclein in bacteria and got enzymatically *O*-GlcNAcylated α-synuclein. The enzymatic *O*-GlcNAcylation also significantly blocked α-synuclein aggregation (Zhang et al., 2017).

## Conclusion and Perspective

**FIGURE 1:**
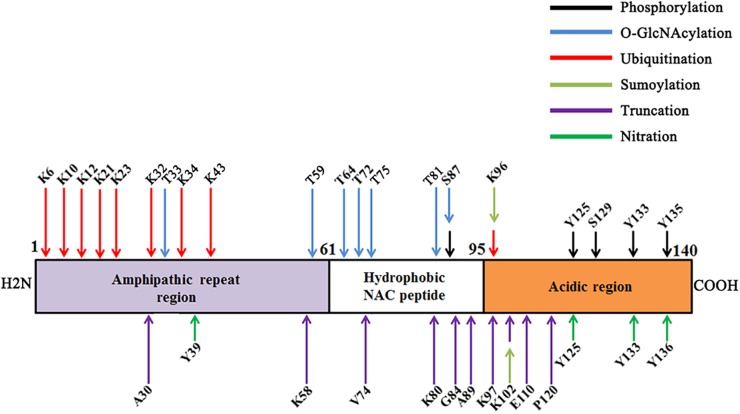
Major post-translational modifications (PTMs) on various amino acids of α-synuclein.

In this review, we have summarized the major PTMs of α-synuclein (Figure 1). Since the presence of PTMs in α-synuclein is able to influence its aggregation and toxicity (Table 1), targeting PTMs may be used to develop novel therapeutic approaches for PD. However, it should be noted that most of the effect of PTMs on the α-synuclein aggregation are carried out *in vitro*; the *in vivo* effect is still elusive. Furthermore, α-synuclein may have multiple different PTMs at the same time *in vivo*; however, the current researches regarding PTMs of α-synuclein are studied individually. The interaction of PTMs of α-synuclein has been widely studied. Phosphorylated α-synuclein has been reported to be one of the target proteins for ubiquitination in synucleinopathies (Hasegawa et al., 2002). Shahpasandzadeh et al. (2014), for the first time, demonstrated an interplay between α-synuclein sumoylation and phosphorylation to control protein turnover. They showed that sumoylation exhibits a protective role against α-synuclein toxicity and inclusion formation in yeast cells (Shahpasandzadeh et al., 2014). There is a complex and dynamic interplay between *O*-GlcNAcylation and phosphorylation (Hart et al., 2007). The interplay between α-synuclein *O*-GlcNAcylation and phosphorylation is still unknown. As mentioned before, neurosin is one enzyme that mediates the truncation of α-synuclein. Kasai et al. (2008) showed that phosphorylated α-synuclein was more resistant to degradation by neurosin than non-phosphorylated α-synuclein. A reciprocal reaction may also occur between other PTMs, which still need further study. Third, it is interesting that, in some cases, the same modification may have a different effect. For instance, phosphorylation at S129 by CKII may promote aggregation, but phosphorylation at S129 by PLK2 promotes degradation. More studies are required to understand the underlying mechanisms for the discrepancy. Lastly, although it is well known that PTMs are regulated by the environmental stimuli, few studies have attempted to use PTMs to link environmental factors and α-synuclein toxicity. Thus, future studies on the PTMs of α-synuclein *in vivo* will help to address these concerns, and improve our understanding surrounding the role of the gene-environment interaction in PD pathogenesis.

**Table 1:** Functional consequences of the major PTMs on α-synuclein.

PTM	Amino acid	Enzyme	Effects	References
Phosphorylation	S129	CKII	Promote aggregation	Fujiwara et al., 2002; Smith et al., 2005
	S129	GRK2	Promote oligomerization	Feany and Bender, 2000
	S129	PLK2	Promote degradation	Oueslati et al., 2013
Ubiquitination	K10, 12, 21, 23, 34, 43, 96	SIAH	Promote aggregation	Lee et al., 2008; Rott et al., 2008
		CHIP	Inhibit aggregation	Shin et al., 2005
			Promote degradation	Shin et al., 2005; Tetzlaff et al., 2008
Sumoylation	K96, 102	PIAS2	Promote aggregation	Rott et al., 2017
			Inhibit degradation	
Truncation	K58, V74	Calpain I	Inhibit aggregation	Mishizen-Eberz et al., 2005
	K80	Neurosin	Inhibit polymerization	Kasai et al., 2008
	K97	Neurosin	Promote polymerization	Kasai et al., 2008
Nitration	Y39, 125, 133, 136	–	Promote aggregation	Hodara et al., 2004
*O*-GlcNAcylation	T72, 75, 81, S87	OGT	Inhibit aggregation	Lewis et al., 2017; Levine et al., 2019


## Author Contributions

All authors participated in designing the concept of this manuscript.

## Conflict of Interest Statement

The authors declare that the research was conducted in the absence of any commercial or financial relationships that could be construed as a potential conflict of interest.
